# Severe Immune Effector Cell-Associated Neurotoxicity Syndrome in a Patient With Multiple Myeloma Treated With Elranatamab

**DOI:** 10.7759/cureus.74902

**Published:** 2024-12-01

**Authors:** Taku Kikuchi, Kodai Kunisada, Kota Sato, Nobuhiro Tsukada, Tadao Ishida

**Affiliations:** 1 Department of Hematology, Japanese Red Cross Medical Center, Tokyo, JPN

**Keywords:** bispecific antibodies, bispecific t cell engager, complication of treatment, elranatamab, immune effector cell-associated neurotoxicity syndrome (icans), relapsed and refractory multiple myeloma

## Abstract

Elranatamab is an effective drug for triple-class-exposed relapsed/refractory multiple myeloma (TCE-RRMM). In the pivotal study, only grade 1 or 2 immune effector cell-associated neurotoxicity syndrome (ICANS) were reported, and the risk factors for immune effector cell-associated neurotoxicity syndrome have not yet been clearly elucidated. This case report documents the first case of grade 4 ICANS in a patient treated with elranatamab, presenting alongside grade 1 cytokine release syndrome (CRS). The patient had a history of cerebral hemorrhage without residual neurological deficits, but its association with ICANS was unclear. High-dose methylprednisolone therapy was required to manage the condition. Magnetic resonance imaging (MRI) findings were negative for new abnormalities, indicating that previous cerebral events could contribute to the risk of ICANS. This case emphasizes the importance of close monitoring of neurological parameters and prompt intervention for patients receiving elranatamab, regardless of a history of neurological conditions or the presence of neurological symptoms at the time of treatment. As more patients are treated with elranatamab, understanding the risk factors for severe immune effector cell-associated neurotoxicity syndrome (ICANS) will be crucial to ensure timely management and improved patient outcomes. Clinicians should be aware of the potential for severe neurotoxicity, even in patients without current neurological deficits, and pay attention to detecting early symptoms and initiate appropriate treatment promptly.

## Introduction

Elranatamab, a bispecific antibody targeting CD3 and B-cell mature antigen (BCMA), has shown promising efficacy in triple-class-exposed relapsed/refractory multiple myeloma (TCE-RRMM) [1-3]. Common adverse events include cytokine release syndrome (CRS) and immune effector cell-associated neurotoxicity syndrome (ICANS). Elranatamab has been associated with lower rates of cytokine release syndrome (CRS) and immune effector cell-associated neurotoxicity syndrome (ICANS) compared to other BCMA-targeted therapies [4,5]. Furthermore, in the pivotal study, there were no reports of grade 4 ICANS among patients receiving elranatamab. Therefore, patients receiving elranatamab are considered to have a low risk of developing ICANS. Herein, we present a previously undocumented case of grade 4 ICANS concomitant with grade 1 CRS following elranatamab administration in a patient with a history of neurological disease, although without any residual symptoms, to raise awareness and emphasize the need for vigilance in similar cases.

## Case presentation

**Figure 1:**
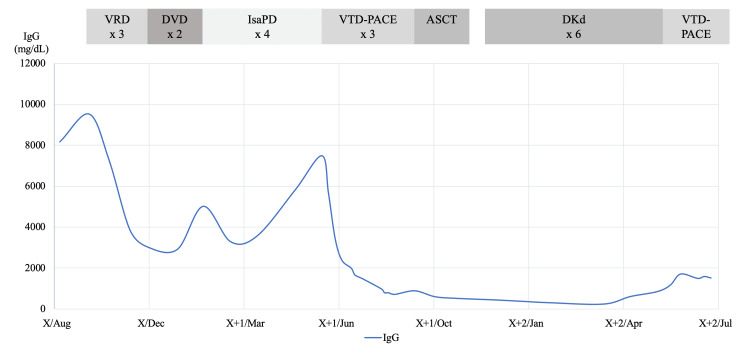
Clinical course from diagnosis to elranatamab administration. VRD; bortezomib, lenalidomide, and dexamethasone, DVD; daratumumab, bortezomib, and dexemethasone, IsaPD; isatuximab, pomalidomide, and dexamethasone, VTD-PACE; bortezomib, thalidomide, dexamethasone, cisplatin, doxorubicin, cyclophosphamide, and etoposide, ASCT; autologous stem cell transplantation, DKD; daratumumab, carfilzomib, and dexamethasone.

A 60-year-old woman was diagnosed with multiple myeloma (revised International Staging System II), positive for deletion 17p and t(4;14), two years prior to initiating elranatamab treatment. The patient had a history of cerebral hemorrhage in the left thalamus 10 years prior to elranatamab administration; however, at the time of the diagnosis of myeloma, imaging revealed only hemosiderin deposition in the left thalamus, with no evidence of residual hemorrhage, and no neurological deficits were observed. Figure 1 shows the clinical course from diagnosis to elranatamab administration.

The patient progressed on bortezomib, lenalidomide, and dexamethasone therapy after three cycles, and the patient also progressed on both daratumumab, lenalidomide, and dexamethasone therapy, as well as isatuximab, pomalidomide, and dexamethasone therapy. A partial response was achieved with bortezomib, thalidomide, dexamethasone, cisplatin, adriamycin, cyclophosphamide, and etoposide therapy (VTD-PACE). Following high-dose melphalan and autologous stem cell transplantation, consolidation therapy with daratumumab, carfilzomib, and dexamethasone (DKd) was administered, but the patient became refractory after six cycles of DKd. She eventually developed TCE-RRMM and penta-drug refractory disease, and treatment was planned with chimeric antigen receptor T-cells (CAR-T), but product manufacturing failed. The patient was started on elranatamab due to the failure of CAR-T manufacturing and disease progression on VTD-PACE, which had been used as bridging therapy to CAR-T. At the time of elranatamab administration, the patient's ferritin level was elevated at 563 ng/mL, but the platelet count remained within the normal range, and no neurological abnormalities were observed. Additionally, lactate dehydrogenase was elevated at 278 U/L, and there were no signs of extramedullary disease. A bone marrow examination was performed at the initiation of the bridging therapy with VTD-PACE, revealing 11% of plasma cells.

**Figure 2:**
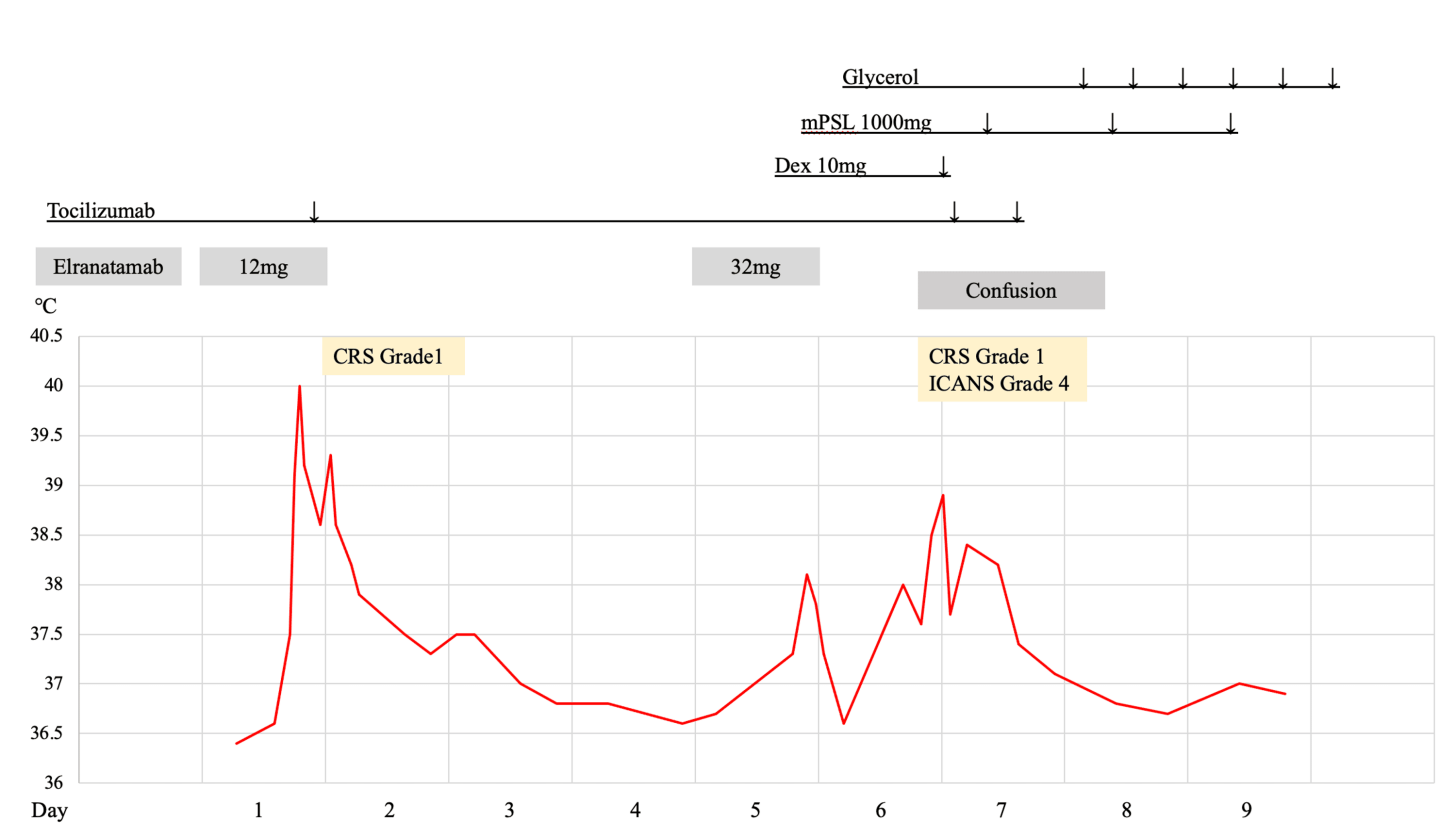
Treatment course following the initiation of elranatamab. mPSL; methylprednisolone, Dex; dexamethasone, CRS; cytokine release syndrome, ICANS; immune effector cell-associated neurotoxicity syndrome.

Figure 2 shows the treatment course after the initiation of elranatamab. 

On the day of the first dose of elranatamab, the patient developed a fever (grade 1 CRS), which was resolved with tocilizumab and acetaminophen. After the second dose, fever recurred and was resolved with acetaminophen alone. At 35 hours post-administration, the patient experienced a headache but maintained an immune effector cell-associated encephalopathy (ICE) score of 10 (Table 1), while remaining alert and communicative.

**Table 1:** ICE score and ICANS grade. ICE; immune effector cell-associated encephalopathy, ICANS; immune effector cell-associated neurotoxicity syndrome.

ICE (immune effector cell-associated encephalopathy)
Orientation to year, month, city, hospital: 4 points (1 point each)
Name 3 objects (eg, point to clock, pen, chair): 3 points (1 point each)
Follow simple commands (eg, Open your mouth) : 1 point
Write a standard sentence : 1 point
Count backwards from 100 by 10 : 1 point
ICANS grade (ICE scoring)
10: No impairment, 7-9: Grade 1, 3-6: Grade 2, 0-2: Grade 3, 0: Grade 4

**Figure 3:**
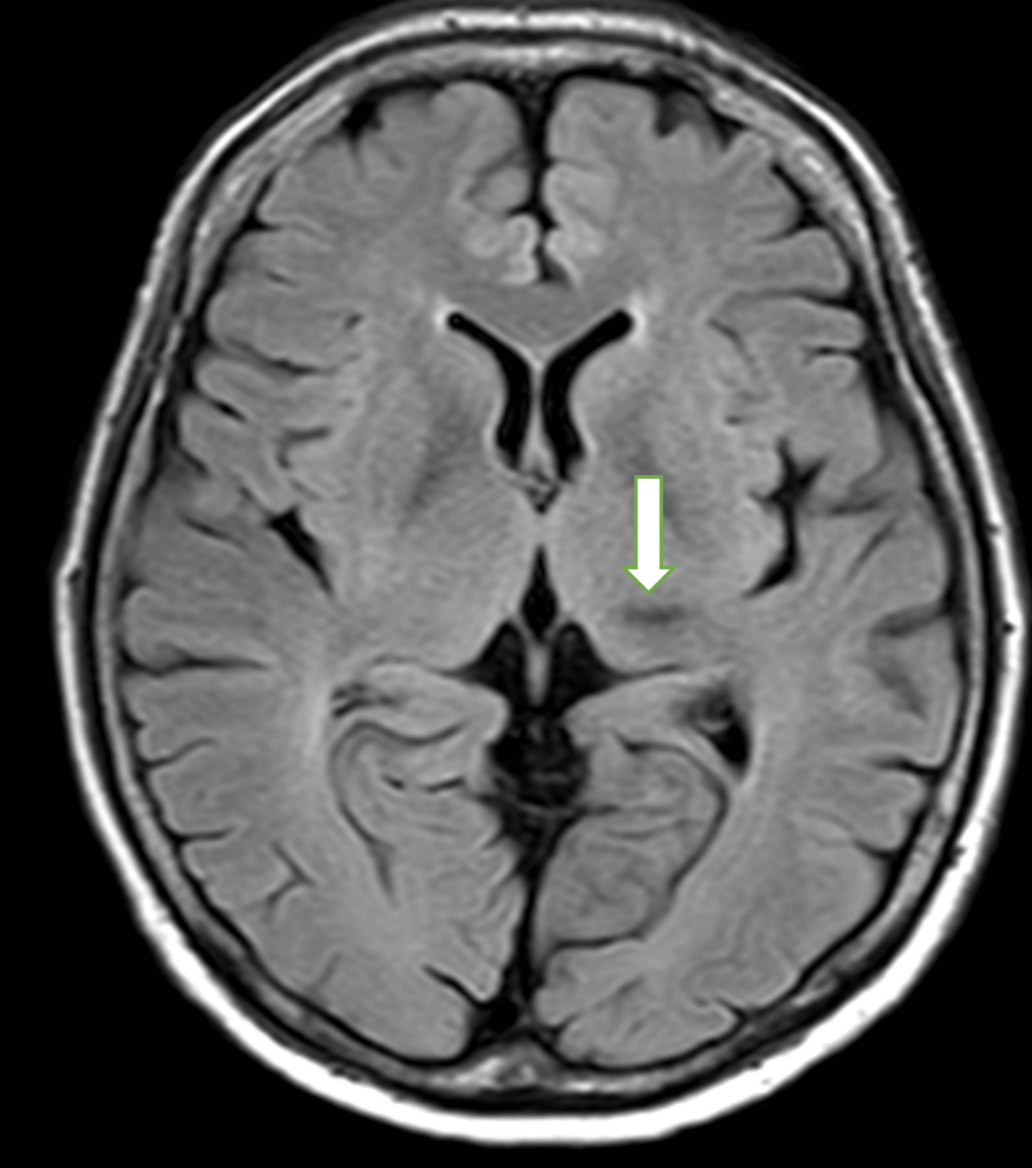
Head magnetic resonance imaging before elranatamab therapy. The arrow indicates hemosiderin deposition in the left thalamus, representing chronic changes from the previous cerebral hemorrhage.

**Figure 4:**
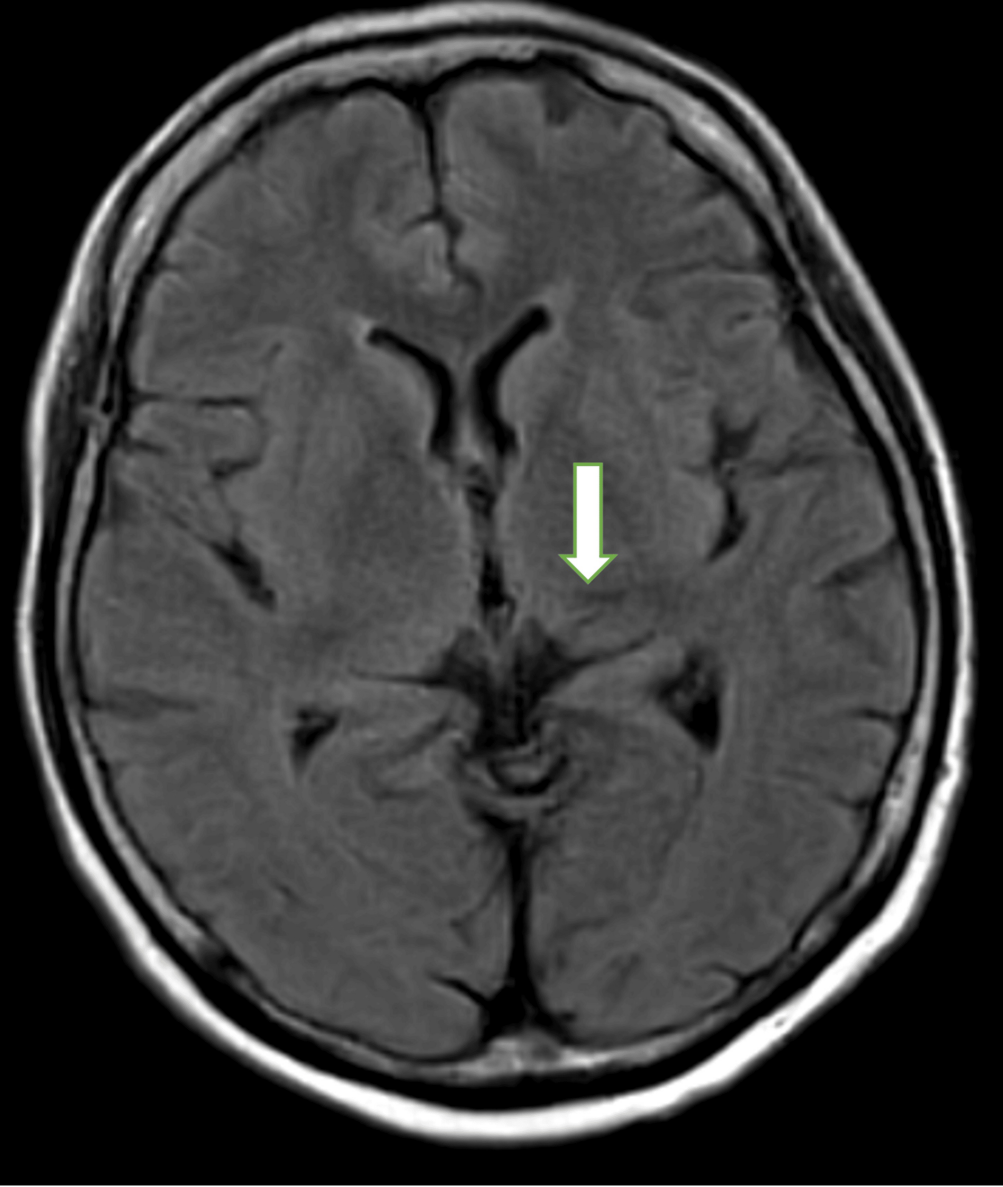
Head magnetic resonance imaging during the state of confusion. The arrow indicates hemosiderin deposition in the left thalamus, representing chronic changes from the previous cerebral hemorrhage.

At 38 hours post-administration, in addition to a severe headache, the patient experienced fever and vomiting, though her level of consciousness remained unaffected at that point. However, at 41 hours post-administration, she became unresponsive and confused. Head magnetic resonance imaging (MRI) taken during the state of confusion when compared to those before elranatamab therapy, showed no evidence of the cause of confusion including cerebral hemorrhage, other than the chronic changes from the prior cerebral hemorrhage and blood test results were unremarkable (Figures 3, 4). 

Evaluation of cerebrospinal fluid pressure and electroencephalography were not possible because of her confusion. An ICE score of 0 led to a diagnosis of grade 4 ICANS according to the criteria of the American Society for Transplantation and Cellular Therapy, and treatment was initiated with dexamethasone 10 mg and levetiracetam [6]. As symptoms persisted 6 hours after dexamethasone administration, a 1000 mg/day methylprednisolone (mPSL) treatment for three days (mPSL pulse) and glycerol administration were performed, because anti-IL-6 antibody siltuximab, which prevents high concentrations of IL-6 in the cerebrospinal fluid, and the IL-1 antagonist anakinra are not available for use in Japan. After 24 hours of initiating mPSL therapy, the fever persisted but the patient's consciousness improved, with an ICE score of 10. Following the mPSL pulse, the dosage of mPSL was tapered, and no recurrence of ICANS was observed. Prior to the onset of ICANS, there were no elevations in ferritin, CRP, or lymphocyte count, and no findings suggested excessive T-cell activation.　The patient achieved a partial response after two cycles and is currently continuing elranatamab therapy on an outpatient basis.

## Discussion

In the MagnetisMM-3 trial, 57.7% of patients exhibited CRS with no cases reaching grade 3 or higher [2]. ICANS occurred in 3.4% of patients, all cases being grade 1 or 2, and were managed with corticosteroids, tocilizumab, and levetiracetam for seizure prophylaxis. In cases of ICANS, imaging studies such as MRI are recommended to differentiate it from conditions like meningitis or cerebral hemorrhage. Lumbar puncture for cerebrospinal fluid analysis and EEG should also be performed. Treatment involves high-dose corticosteroids, and antiepileptic drugs are recommended for seizure management. If signs of increased intracranial pressure are present, the administration of mannitol may be considered. However, the significance of VV/P shunting remains unclear [7].

Our case, which presented with severe headache and confusion with an ICE score of 0, necessitated high-dose mPSL therapy and showed improvement within 24 hours. To our knowledge, this is the first reported case of severe ICANS in a patient with multiple myeloma treated with elranatamab. In the pivotal study of teclistamab, a bispecific antibody targeting BCMA, ICANS was observed in 14.5% of cases, with one case of grade 4 neurotoxicity attributed to bacterial meningitis during cycle 7 [8]. In contrast, a real-world multicenter report involving 110 patients indicated that ICANS occurred in 11% of patients, with grade 3 or higher ICANS observed in five cases (4.5%), including one grade 5 case resulting in death [9]. The deceased patient exhibited steroid-resistant ICANS complicated by hemophagocytic syndrome. Another single-center study reported two cases of grade 3 ICANS following teclistamab administration, one of which resulted in death due to complications [10]. In these cases, anakinra was administered in addition to steroids, but one patient ultimately succumbed. Neither of the two cases showed evidence of CNS involvement, although one had a history of transient ischemic attack (TIA). Notably, the current case did not exhibit any neurological sequelae; however, the patient had a history of microhemorrhage from 10 years prior to elranatamab administration. There were no neurological sequelae, and MRI images both before and after elranatamab treatment showed only chronic changes after the cerebral hemorrhage, specifically hemosiderin deposition in the left thalamus. 

The mechanisms of ICANS are not fully understood. However, several potential explanations have been proposed. It is often associated with cytokine release syndrome (CRS), which leads to the activation of T cells and the release of inflammatory cytokines like interleukin-6 (IL-6), IL-1, and interferon-γ. These cytokines can penetrate the CNS and cause neurotoxicity. They may also disrupt the blood-brain barrier (BBB), increasing its permeability and allowing inflammatory molecules and immune cells to enter the brain. Furthermore, excessive cytokine release can damage endothelial cells, potentially resulting in brain edema or microhemorrhages. These processes collectively contribute to the development of ICANS. In addition, BCMA CAR-T therapies have a higher incidence of cytokine release syndrome compared to BCMA bispecific antibodies (BsAb), possibly due to higher cytokine production [11]. This may be one of the reasons why the incidence of ICANS is higher with CAR-T therapy compared to BsAb therapy. However, further investigation is needed.

In our case, the association between a history of cerebral hemorrhage and the development of ICANS remains unclear. However, a pre-existing neurologic condition is considered a putative risk factor for ICANS, at least in CAR-T therapy, and whether this can be extrapolated to BsAb therapy requires further investigation through accumulated cases [12]. Reports on the treatment outcomes and adverse events of elranatamab remain limited, and as the number of patients receiving elranatamab increases, there is a possibility that cases of severe ICANS may emerge. Therefore, while the risk of ICANS has so far been considered low, close monitoring of ICE scores and other neurological parameters after elranatamab administration is crucial for the early detection and timely intervention of ICANS.

## Conclusions

Although elranatamab has been associated with lower rates of CRS and ICANS compared to other BCMA-targeted therapies, its potential for severe ICANS should be considered. Further investigation through the accumulation of cases is needed to clarify the association between a history of neurological conditions and the development of ICANS after elranatamab administration.
